# Computational Modeling Study of the Binding of Aging and Non-Aging Inhibitors with Neuropathy Target Esterase

**DOI:** 10.3390/molecules28237747

**Published:** 2023-11-24

**Authors:** Wenxiong Wu, Pan Wang

**Affiliations:** Shenzhen Key Laboratory of Steroid Drug Discovery and Development, School of Medicine, The Chinese University of Hong Kong, Shenzhen 518172, China

**Keywords:** computational modeling, neuropathy target esterase, molecular docking

## Abstract

Neuropathy target esterase (NTE) is a serine hydrolase with phospholipase B activity, which is involved in maintaining the homeostasis of phospholipids. It can be inhibited by aging inhibitors such as some organophosphorus (OP) compounds, which leads to delayed neurotoxicity with distal degeneration of axons. However, the detailed binding conformation of aging and non-aging inhibitors with NTE is not known. In this study, new computational models were constructed by using MODELLER 10.3 and AlphaFold2 to further investigate the inhibition mechanism of aging and non-aging compounds using molecular docking. The results show that the non-aging compounds bind the hydrophobic pocket much deeper than aging compounds and form the hydrophobic interaction with Phe1066. Therefore, the unique binding conformation of non-aging compounds may prevent the aging reaction. These important differences of the binding conformations of aging and non-aging inhibitors with NTE may help explain their different inhibition mechanism and the protection of non-aging NTE inhibitors against delayed neuropathy.

## 1. Introduction

Neuropathy target esterase (NTE), also called patatin-like phospholipase domain-containing protein 6 (PNPLA6), is the sixth member of the protein family of patatin domain lipid hydrolase [1]. NTE is a serine hydrolase with phospholipase B activity [2], which has both phospholipase A1 and A2 activities [3] and could deacetylate both acyl bonds in phosphatidylcholine (PtdCho) [4,5,6]. NTE is important in maintaining the stability of membrane phospholipids [6,7,8,9]. NTE plays important roles in vasculogenesis [10] and axon maintenance [11].

In addition, NTE is involved in chronic neuropathies induced by organophosphorus compounds (OP) and other NTE inhibitors [12]. OP compounds are used widely in the chemical, agriculture, or pharmaceutical industries [6,13]. Aging inhibitors could cause delayed neurotoxicity, characterized by a distal degeneration of axons in the central nervous system (CNS) and peripheral nervous system (PNS) [6,12].

There are two types of NTE inhibitors. One is aging inhibitors, which include certain phosphates, phosphonates, and phosphoramidates [12,14,15,16,17]. The aging of NTE is the loss of reactivity of NTE [12,18,19,20]. The other type of NTE inhibitors is non-aging inhibitors, which usually include phosphinates, sulfonates, and carbamates. Non-aging inhibitors can inhibit NTE activity but would not cause aging on NTE due to the reactivation of NTE. The aged NTE could not be reactivated [12]. The non-aging inhibitors will not produce delayed neuropathy and can protect the animals from neurotoxicity induced by aging inhibitors [6,12]. For example, non-aging inhibitor phenylmethylsulfonyl fluoride (PMSF) can protect against the delayed neuropathy induced by tri-ortho-cresyl phosphate (TOCP) in hens [21] and protect against diisopropylfluorophosphate (DFP)-induced delayed neuropathy in cats [22,23] and hens [24]. 

A previous study reported the construction of a homology model of NTE esterase domain (NEST) using the template protein patatin isoform 17 (Pat17) to study the catalytic mechanism of NTE [25]. However, the identity between the sequences of NTE and Pat17 is rather low (18%) [25]. Moreover, little was known about the different binding of aging/non-aging inhibitors with NEST.

This study aims to construct a more precise model of NEST using templates with higher identity by using both AlphaFold2 and MODELLER 10.3. In addition, the possible mechanism of inhibition by various aging and non-aging inhibitors was investigated by using molecular docking. We revealed important differences of the binding conformations of aging and non-aging inhibitors with NTE, which may explain their different inhibition mechanism and the protection of non-aging NTE inhibitors against delayed neuropathy.

## 2. Results 

### 2.1. Construction and Validation of the NEST Models

**Figure 1:**
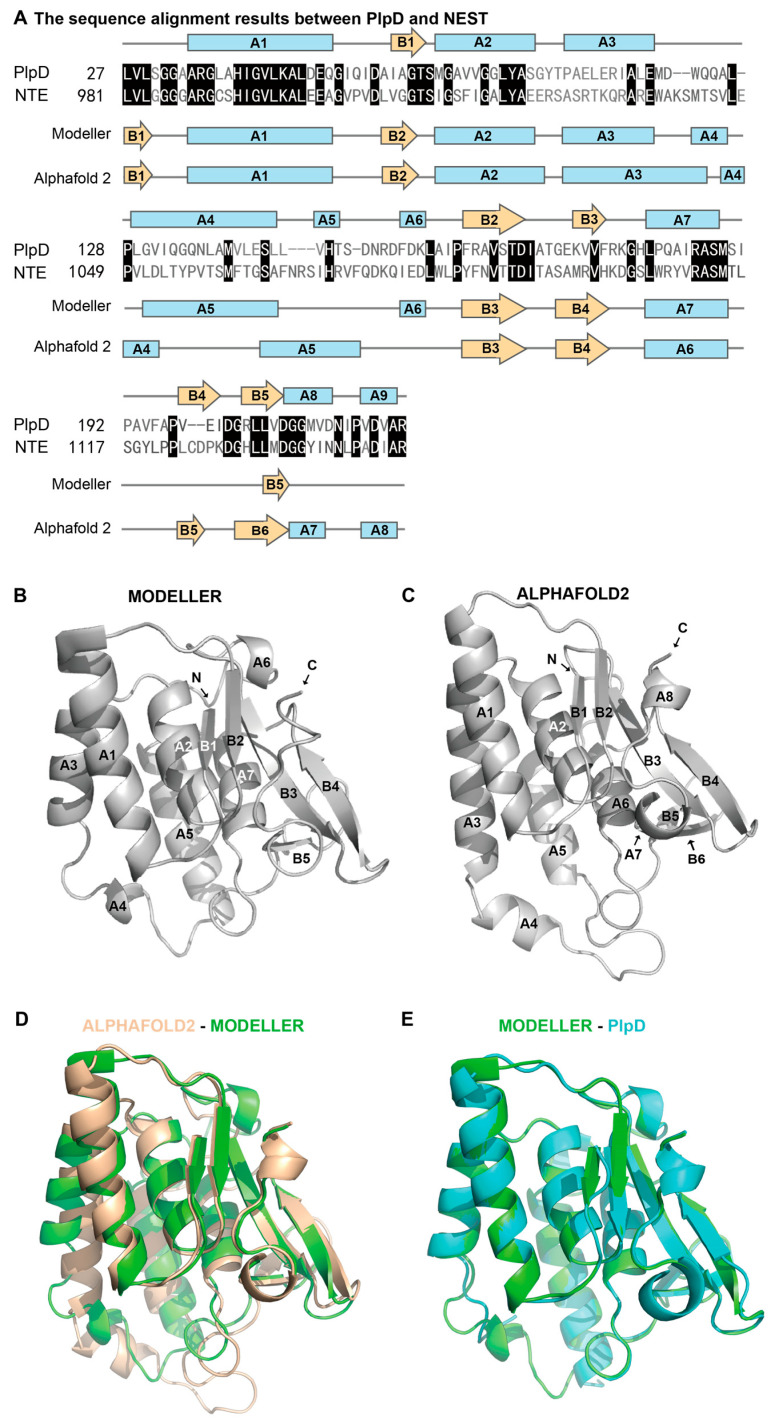
The constructed NEST models. (**A**) The sequence alignment results between PlpD and NEST. Identical residues have been boxed in the black color. Secondary structures of PlpD (shown on the top of the sequence) and two NEST models built by MODELLER 10.3 and the AlphaFold2 (shown below the sequence) are displayed. β-strands are shown as arrows colored in cream and α-helices are shown as rectangles colored in blue. (**B**,**C**) The overall structure models of NEST built by MODELLER 10.3 (**B**) and AlphaFold2 (**C**). (**D**,**E**) The overlay of AlphaFold model and MODELLER model (**D**) and the overlay of the MODELLER model with the PlpD structure (**E**). The PlpD structure, the MODELLER model, and the AlphaFold model are colored blue, green, and cream, respectively.

Homology modeling was used to construct the 3D models of the NTE catalytic domain NEST with homologous template protein. A BLAST search demonstrated that the native PlpD (PDB ID: 5FYA) shares 30.46% identity with the NEST sequence and the E value is 2 × 10^−21^ (Figure 1A). This sequence identity is much higher than that of the protein template used in the previous study, which used Pat17 with only 18% sequence identity with the NEST as the template protein to build NEST models [25]. The 3D structure analysis of NEST built by MODELLER10.3 indicates that the overall structure of NEST contains seven α-helices (named A1–A7) and five β-strands (named B1–B5) (Figure 1C). In addition, the model of NEST by AlphaFold2 contains eight α-helices (named A1–A8) and six β-strands (named B1–B6) with high model confidence (the predicted local-distance difference test (pLDDT) > 90) (Figure 1B).

The overlay between the template PlpD (PDB ID: 5FYA) and the NEST homology model built with MODELLER 10.3 indicates that they have high similarity with RMSD 0.141 Å (Figure 1E). The alpha-carbon RMSD value between the homology model built by MODELLER 10.3 and AlphaFold2 is 1.30 Å, which indicates that these two models are very similar to each other (Figure 1D,E).

**Figure 2:**
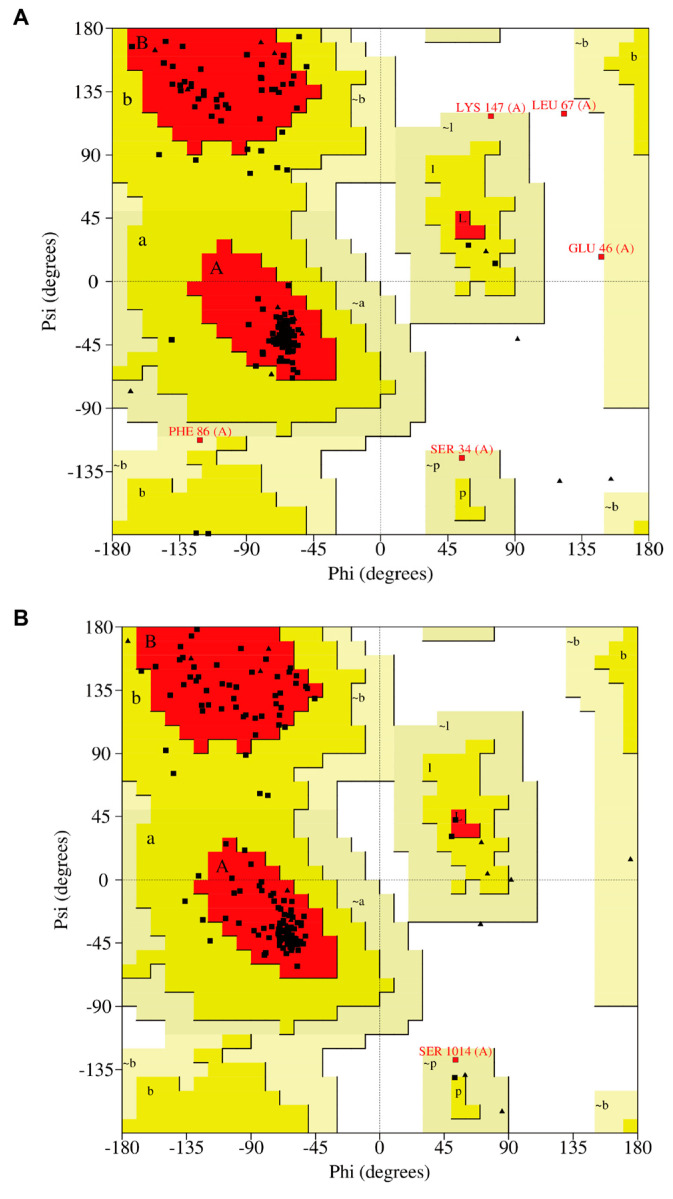
The assessment of the models of NEST. (**A**) Ramachandran Plot and Procheck result of the homology model built by MODELLER 10.3, in which 98.6% of the amino acid residues are in the reasonable zone. (**B**) Ramachandran Plot and Procheck result of the model built by the AlphaFold2, in which 100% of the amino acid residues are in the reasonable zone. In this Ramachandran Plot, the “most favored” regions are colored in red, “additional allowed” regions are colored in deep yellow, and the “generously allowed” regions are colored in light yellow while “disallowed” regions are colored in white. One black point represents one residue. Triangles indicate the glycine residues while rectangles indicate other residues. “A”, “a” and “~a” indicate the “most favored”, “additional allowed” and “generously allowed” regions of right-handed alpha helices, respectively. “B”, “b” and “~b” indicate the “most favored”, “additional allowed” and “generously allowed” regions of beta sheets, respectively. “L”, “l” and “~l” indicate the “most favored”, “additional allowed” and “generously allowed” regions of left-handed alpha helices, respectively. “p” and “~p” indicate the regions of “additional allowed” and “generously allowed” epsilon.

The NEST models were evaluated by SAVES v6.0. The Ramachandran results for the models built with MODELLER 10.3 and AlphaFold2 are shown in Figure 2A,B, respectively, which shows that 98.6% of the amino acid residues are in the reasonable zone (86.4% of the residues in the “most favored” regions, 10.0% in the “additional allowed” regions, and 2.1% in the “generously allowed” regions). For the model built by the AlphaFold2, 100% of the amino acid residues are in the reasonable zone (91.4% of the residues in the “most favored” regions, 7.9% in the “additional allowed” regions, and 0.7% in the “generously allowed” regions). Therefore, both models were validated as acceptable models, and the model built by AlphaFold2 is relatively better and more reasonable compared to the model of MODELLER 10.3.

### 2.2. The NEST Active Site Structures

**Figure 3:**
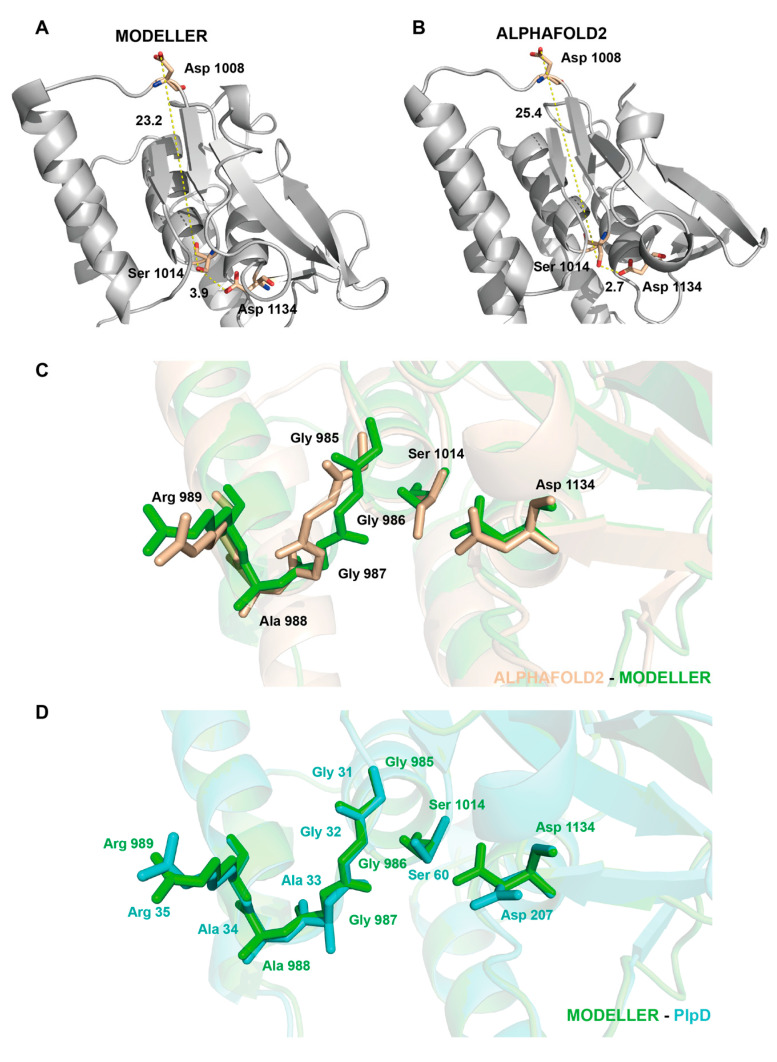
The overlay of the overall structures of NEST models and important active residues. (**A**,**B**) The enlarged view of the predicted active site of NEST. Important residues, such as Ser1014, Asp1008, and Asp1134 are shown as sticks. Distances between Ser1014 Oγ and Asp1008 O_δ2_ and Ser966 Oγ and Asp1134 O_δ2_ are shown as dotted lines. (**C**,**D**) The overlay of important residues (including Ser1014, Asp1134, and oxyanion hole residues between AlphaFold model and MODELLER model (**C**) and between the MODELLER model and the PlpD (**D**). The PlpD structure, the MODELLER model, and the AlphaFold model are colored blue, green, and cream, respectively.

In the NEST model built with MODELLER 10.3 (Figure 3A), the overlay of the active sites of the two models including Ser1014, Asp1134, and oxyanion hole residues show that these key residues have very similar conformations (Figure 3C,D). Ser1014 and Asp1134 are located in the two conserved loops B2–A2 and B5–C terminal, respectively, and Ser1014 Oγ is located 3.9 Å from the Asp1134 Oδ_2_. In the model built with AlphaFold2 (Figure 3B), Ser1014 and Asp1134 are located in the conserved loops B2–A2 and B3–B4, and Ser1014 Oγ is located 2.7 Å from the Asp1134. Early studies have shown that Asp1008 is key to the activity of phenyl-valerate hydrolase in NEST with site-directed mutagenesis [26]. However, in our model, the distance between Asp1008 Oδ_2_ and Ser1014 Oγ is 23.2 Å in the model built with MODELLER 10.3 and 25.4 Å in the model built with AlphaFold2. Therefore, this distance is too far for Asp1008 to play a direct role in catalysis. 

**Figure 4:**
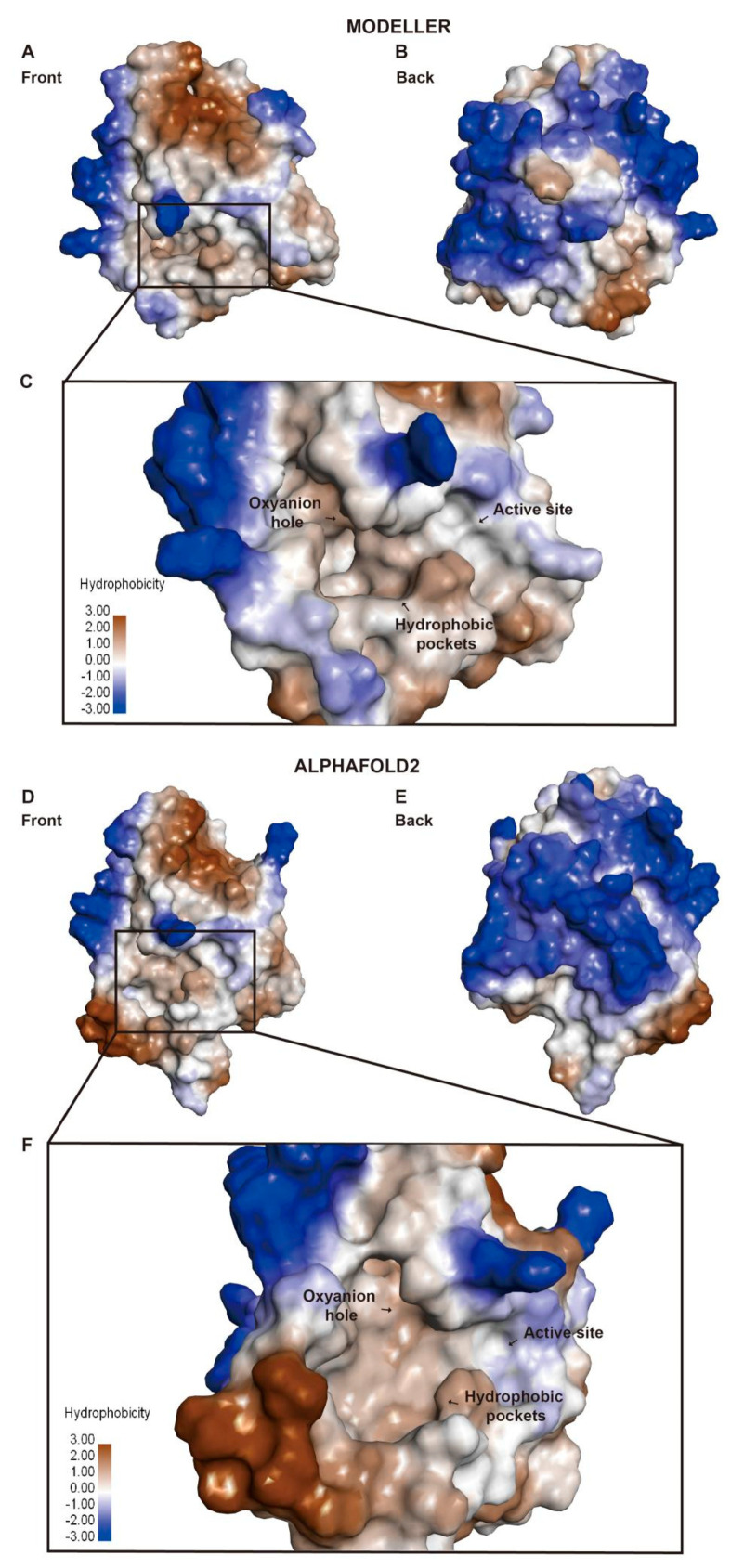
Hydrophobicity analysis of the NEST models built by MODELLER 10.3 and AlphaFold2. Hydrophobic regions are shown in brown and hydrophilic regions in blue. (**A**) The front view of the NEST homology model by MODELLER 10.3. (**B**) The back view of the NEST homology model by MODELLER 10.3. (**C**) The zoom-in view of the active site by MODELLER 10.3. (**D**) The front view of NEST model by AlphaFold2. (**E**) The back view of the NEST model by AlphaFold2. (**F**) The zoom-in view of the active sites by AlphaFold2.

Hydrophobicity analysis of the NEST ligand binding pocket built with both MODELLER 10.3 and AlphaFold2 shows that there are two vital areas in the NEST ligand binding site (Figure 4). One is the hydrophilic pocket consisting of active sites Ser1014-Asp1134 and oxyanion hole mainly consisting of Gly986-Gly987-Ala988-Arg989 (Figure 4C,F). The other is the hydrophobic pocket (Figure 4C,F) made up of several nonpolar amino acids and aromatic amino acids, for example Leu1116, Met1114, and Phe1066. 

**Figure 5:**
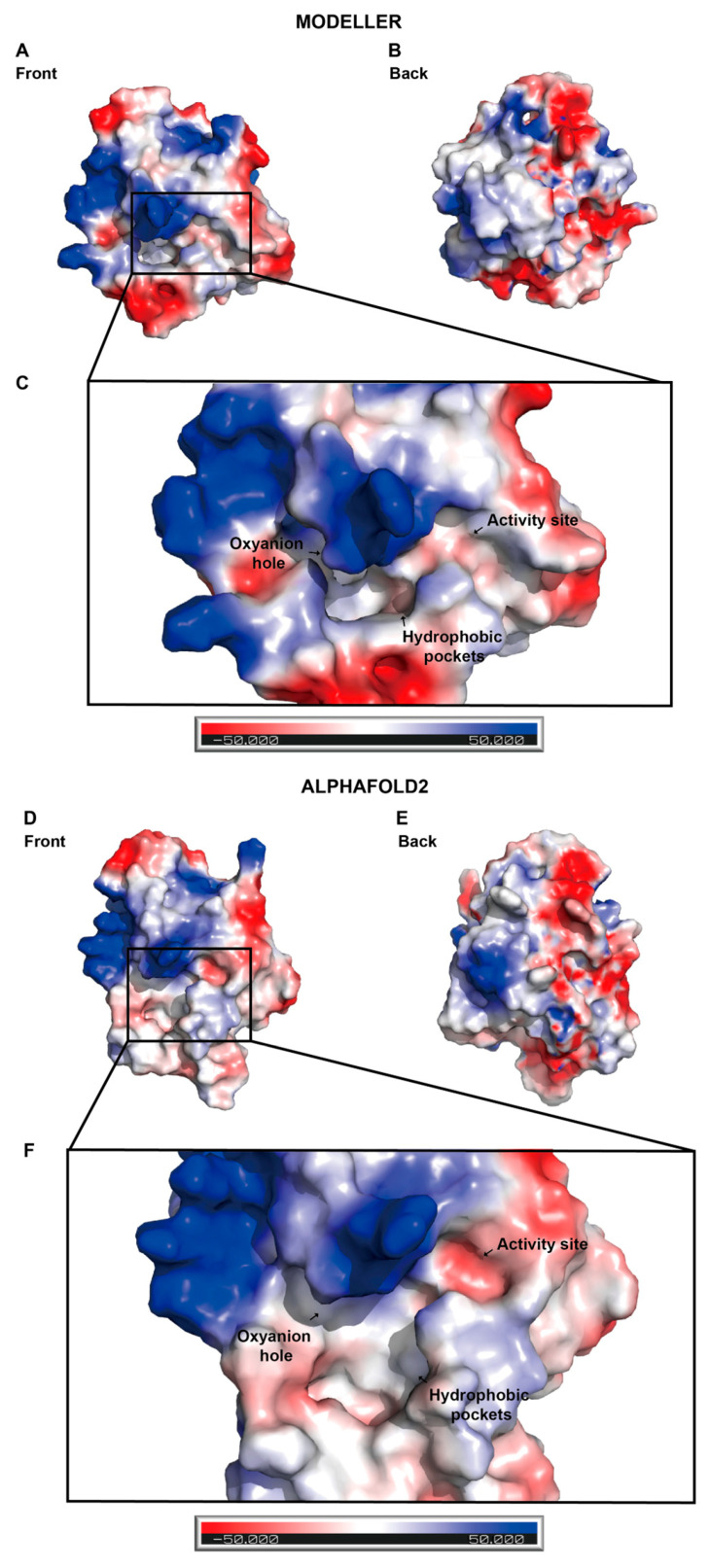
Electrostatic surface potential of the NEST models built by MODELLER 10.3 and AlphaFold2. Acidic regions are shown in red and basic regions in blue. (**A**) The front view of NEST homology model by MODELLER 10.3. (**B**) The back view of NEST homology model by MODELLER 10.3. (**C**) The zoom-in view of the active site by MODELLER 10.3. (**D**) The front view of NEST model by AlphaFold2. (**E**) The back view of NEST model by AlphaFold2. (**F**) The zoom-in view of the active site by AlphaFold2.

The surface of NEST can be divided into two areas, i.e., the hydrophilic area and the hydrophobic area (Figure 4 and Figure 5). In Figure 5, the red, blue, and white colors indicate that the area is negatively charged, positively charged, and neutral, respectively. It is possible that the hydrophilic area of NEST is exposed to the cytosol while the opposite side is located in the ER membrane. The ligand binding pocket is mostly located in the hydrophobic area surrounded by the hydrophilic area (Figure 4 and Figure 5). 

### 2.3. Binding of Aging and Non-Aging Inhibitors with NEST

Then, we studied the binding of five inhibitors and one artificial substrate phenyl valerate (PV) with NEST. The five inhibitors include three aging inhibitors, i.e., Mipafox, DFP, and 2-(ortho-cresyl)-4H-1,2,3-benzodioxaphosphoran-2-one (CBDP), as well as two non-aging inhibitors, i.e., phosphinic acid, PMSF. These ligands were selected and classified according to previous studies [6,12,27]. The CDOCKER interaction energy values of these six ligands with NEST are shown in Table 1. Two aging inhibitors CBDP and mipafox have similar binding energy as the substrate PV with NEST, indicating that their binding affinity with NEST is similar to the substrate PV. Non-aging inhibitors PMSF, phosphinic acid, and aging inhibitor DFP have relatively lower binding affinity with NEST compared to the substrate PV as indicated by their higher CDOCKER energy values. 

**Figure 6:**
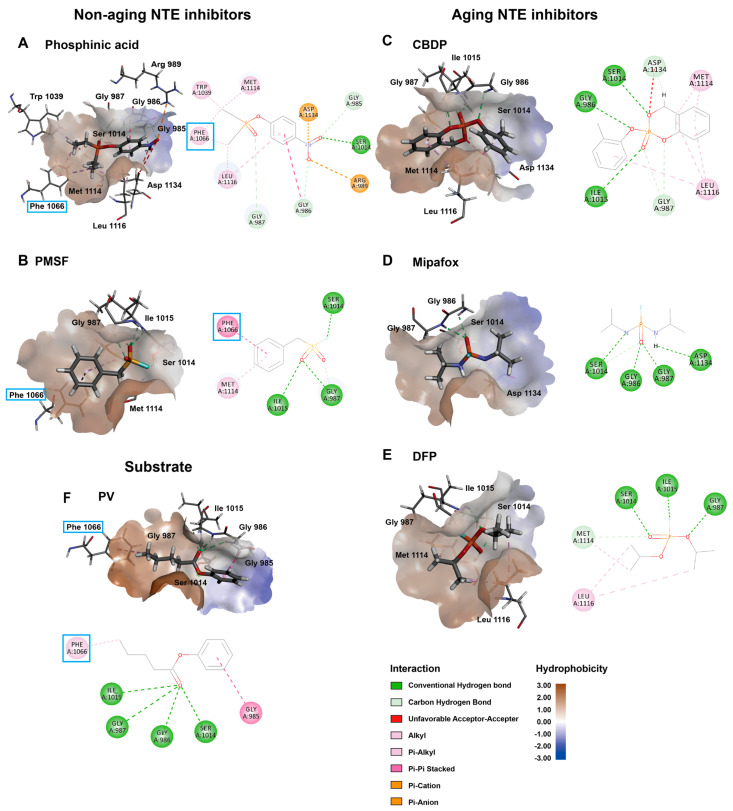
Docking results between NEST and different ligands. (**A**) Phosphinic acid. (**B**) PMSF. (**C**) CBDP. (**D**) Mipafox. (**E**) DFP. (**F**) PV. The ligands are shown in stick and surface of the protein is shown as surface and colored by hydrophobicity. Phosphinic acid and PMSF are non-aging inhibitors while CBDP, Mipafox, and DFP are aging compounds. PV is an artificial substrate of NTE. Dash lines with different colors in the 2D maps indicate different types of interactions, which are shown in the figure. Phe1066 is highlighted with a blue box.

The binding interaction of the inhibitors and NEST predicted by molecular docking is shown in Figure 6. Most hydrogen bonds are formed with the oxyanion hole and Ser1014-Asp1134. Residue Ser1014 forms hydrogen bonds with all the ligands. Compared to Ser1014, Asp1134 is less involved in hydrogen bond formation with ligands in both aging and non-aging inhibitors (Table 2). 

Moreover, our results show that the key oxyanion hole residues Gly986 and Gly987 are involved in the formation of hydrogen bonds with inhibitors and an artificial substrate (PV). It indicates that those two important residues may stabilize and fix the phosphate group of the ligands at the position to stabilize the negative charge of the phosphate group (Table 2 and Figure 6). Gly987 forms hydrogen bonds with all the ligands, which indicates that Gly987 is essential to all ligands which can bind to NEST.

Furthermore, the non-polar amino acids in the hydrophobic pocket such as Met 1114 and Leu1116 form the hydrophobic interactions with the alkyl group of the corresponding ligands, which indicates that these hydrophobic amino acids are also important for the binding of ligands to NEST. It is of note that Phe1066 in the NEST binding site has hydrophobic interaction with substrate PV and non-aging inhibitors, but not with aging inhibitors. This result indicates that Phe1066 in the hydrophobic pocket may play a unique role in the inhibition mechanism of non-aging inhibitors. 

**Figure 7:**
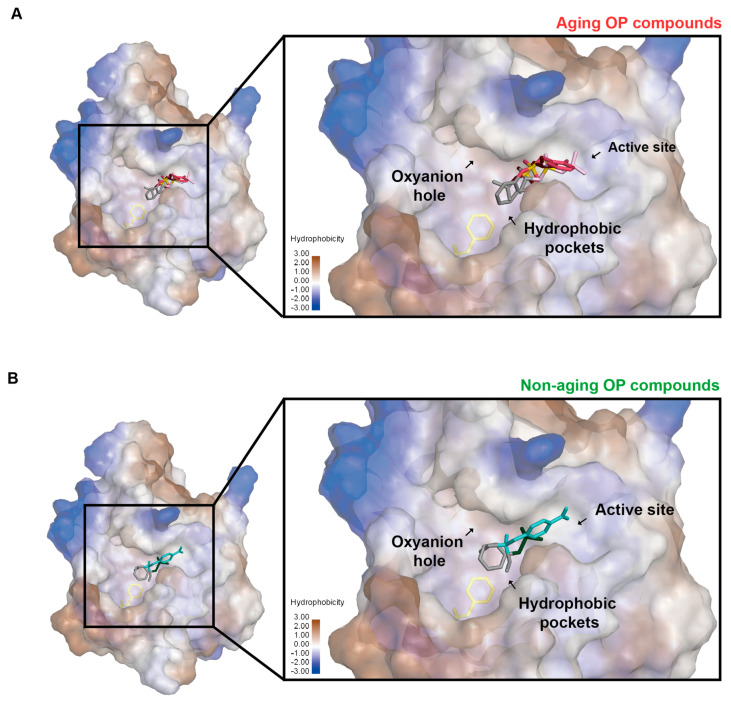
Overlay of the docking results of NEST with non-aging and aging inhibitors. (**A**) Binding of three aging inhibitors CBDP (light red), Mipafox (pink), and DFP (dark red) with the NEST model. (**B**) Binding of two non-aging inhibitors PMSF (dark green), and phosphinic acid (light green) with the NEST model. The protein is shown in surface and colored by hydrophobicity. The phosphate groups of the inhibitors are colored in yellow and the alkane groups of the inhibitors are colored in gray.

The docking results show that the non-aging inhibitors have a different binding interaction with NEST compared to aging inhibitors. Overall, the non-aging inhibitors bind the hydrophobic pocket much deeper than aging inhibitors and non-aging inhibitors and the substrate are closer to Phe1066 and form the hydrophobic interaction with Phe1066 (Figure 7, Table 3). The average values of the shortest distance to Phe1066 for aging inhibitors and non-aging inhibitors (plus the substrate) are 6.0 ± 0.2 Å and 4.7 ± 0.1 Å, respectively (Table 3). There is a statistical significance between these two groups (*p* = 0.001 < 0.05). These differences in binding interactions between non-aging and aging inhibitors may contribute to the differences in their inhibition mechanism for NEST.

## 3. Materials and Method

### 3.1. Modeling of NEST 

The protein sequence of neuropathy target esterase (isoform 2, primary accession: Q8IY17) was obtained from Uniprot (available at https://www.uniprot.org/ (accessed on 24 October 2022)). NTE esterase domain (NEST) is the catalytic domain with amino acids from 727 to 1216. Then, the amino acid sequence of NEST was input into the National Center for Biotechnology Information (NCBI, available at https://www.ncbi.nlm.nih.gov/ (accessed on 24 October 2022)) database and the basic local alignment search tool (BLAST, available at https://blast.ncbi.nlm.nih.gov/Blast.cgi (accessed on 24 October 2022)) was used to search the similar protein sequences within sequence databases using blastp (protein-protein BLAST). In the Research Collaboratory for Structural Bioinformatics (RCSB) Protein Data Bank (PDB) database, there are in total three proteins that had similar primary structures with NEST, namely, type III effector protein (ExoU) from *Pseudomonas fluorescens A506* (PDB ID: 4QMK), vacuolar protein sorting inhibitor protein D (VipD) from *Legionella pneumophila* (PDB ID: 4AKF), and the bacterial patatin-like proteins (PlpD) from *Pseudomonas aeruginosa PAO1* (PDB ID: 5FYA). The PlpD (PDB ID: 5FYA) was selected as the modeling template because of the high identity (30.46%) with the NEST sequence. 

MODELLER 10.3 (https://salilab.org/modeller/ (accessed on 24 October 2022)) was used for constructing the homology model of NEST. The alignment was automatically generated by MODELLER 10.3. In total, 100 models were built, and the conformation with the lowest discrete optimized protein energy (DOPE) value was selected as the model for verification and analysis [28]. 

Another model of NEST was predicted by using the AlphaFold Monomer v2.0 pipeline from AlphaFold Protein Structure Database (https://alphafold.ebi.ac.uk/ accessed on 24 October 2022) developed by DeepMind and EMBL-EBI [29]. The two models were evaluated with the residual percentage in a Ramachandran plot and Verify3D score via SAVES v6.0 (https://saves.mbi.ucla.edu/ (accessed on 24 October 2022)). Pymol (DeLano Scientific LLC, San Carlos, CA, USA) was utilized to visualize the tertiary structure. 

### 3.2. Molecular Docking

The three-dimensional chemical structure of ligands (.sdf files) was obtained from PubChem (https://pubchem.ncbi.nlm.nih.gov/ (accessed on 24 October 2022)). DiscoveryStudio2019 v19.1.0.18287 (Dassault Systemes Biovia, San Diego, CA, USA) was used for molecular docking for NEST. The model generated by AlphaFold2 was used for the molecular docking because this model has better assessment results based on Ramachandran plots and Procheck analysis. 

The NEST protein and the small ligands were cleaned and prepared using DiscoveryStudio2019 v19.1.0.18287. All ligands were processed by using the DiscoveryStudio2019 v19.1.0.18287 ligand preparation module. The coordinates for the center of the docking site were x: 29.839702, y: −4.038964, and z: −22.620865 with a radius of 10 Å. Flexible docking was used as the docking protocol. Five important residues (Gly986, Gly987, Ala988, Arg989, Ser1014, Asp1134) were allowed to change conformations according to the study of human cytosolic phospholipase A_2_ (cPLA_2_) [30].

The molecular docking conformations were evaluated according to their docking energy. For each conformation, we chose the smallest CDOCKER energy for each ligand as the best conformation, since a lower energy value indicates a more feasible binding [31]. CDOCKER energy calculates the intermolecular energy between the ligand and receptor. Pymol and DiscoveryStudio2019 v19.1.0.18287 were used to perform electrostatic surface potential and hydrophobicity analysis, respectively.

## 4. Discussion

In this study, more precise 3D models built based on templates with higher sequence identity were constructed for the NTE catalytic domain compared to the previous study [25]. The models were built by using both MODELLER 10.3 and the AlphaFold2. Compared to the previous study which used the protein template Pat17 with a low sequence identity (18%) [25], our study used the template with 30% sequence identity. 

In addition, we carried out molecular docking analysis of several representative NTE inhibitors. We find that the hydrophobic pocket residues below the oxyanion hole participates in the stabilization of NTE inhibitors, which has not been reported in the previous study. In addition, we find that the binding interaction with the non-aging inhibitors and aging inhibitors are different, and the hydrophobic pocket, especially Phe1066, plays an important role in the binding of non-aging inhibitors, but not in the binding of aging inhibitors. 

The hydrophobicity analysis shows that the ligand binding pocket of NEST is generally hydrophilic and located in the hydrophobic side. The docking result shows that there are three important components of the binding site which recognize inhibitors (Figure 6 and Table 2). They are the catalytic dyad (Ser1014-Asp1134), hydrophobic pocket residues (Phe1066, Met1114, Leu1116), and oxyanion hole residues (Gly986, Gly987). The polar part of the ligands, i.e., the phosphate group tends to fall into the hydrophilic pocket of the NEST binding site, while the alkane group of the ligands tends to be trapped by the hydrophobic amino acids. The oxyanion hole plays an important role in stabilizing the phosphate group (Figure 6, Table 2). 

Notably, we find that the binding of aging inhibitors to NEST is different compared to non-aging inhibitors. Firstly, compared to Ser1014, Asp1134 seems to play a less essential role in the binding of NTE inhibitors. In addition, Phe1066 directly interacts with the substrate (PV) and non-aging inhibitors but not with aging inhibitors (Figure 7). The overlay docking results among non-aging inhibitors and aging inhibitors indicate that the non-aging inhibitors bind deeper in the hydrophobic pocket than aging inhibitors and form hydrophobic interaction with Phe1066, which is the key feature of binding of non-aging inhibitors (Figure 7). Furthermore, the distance between the aging inhibitors and Phe106 is significantly longer than that of the non-aging inhibitors and the substrate (Table 3). Therefore, non-aging inhibitors may be trapped in the hydrophobic pocket, which may prevent the aging reaction. 

In addition, the binding of non-aging inhibitors may prevent the subsequent binding of aging inhibitors. This may help explain the phenomena that the non-aging inhibitors can prevent delayed neuropathy when they are given before aging compounds, but cannot prevent delayed neuropathy when they are given after aging compounds [32]. When non-aging inhibitors are given before aging inhibitors, they may block the binding of aging inhibitors and NTE would not age, but when non-aging inhibitors are given after aging inhibitors, they cannot block the binding of aging inhibitors and cannot prevent NTE aging.

It is of note that the binding of the substrate PV to NEST is more similar to the non-aging inhibitors than to the aging inhibitors (Figure 6, Table 2). PV forms hydrophobic interaction with Phe1066 and there is no interaction with Asp1134. The distance of PV to Phe1066 is shorter compared to aging inhibitors (Table 3). This may explain why the catalytic reaction with the substrate usually does not result in the aging of NEST. 

Early studies have shown that Asp1008 is key to the activity of phenyl-valerate hydrolase in NEST with site-directed mutagenesis [26]. However, our study shows that Asp1008 is at a far distance from the active site and is not likely to directly participate in the catalytic process. One possible explanation for the role of Asp1008 is that the mutation of this residue will change its nearby structure and folding of the anti-parallel β sheet. 

Further computational studies such as molecular dynamics simulations may be used to further test the current hypothesis. Molecular dynamic simulation could evaluate the dynamics of the binding complexes of NEST with different inhibitors and provide more information on the interaction of inhibitors with NEST. Computation using quantum mechanics may also help understand the reaction mechanism of aging inhibitors or substrates with the amino acids in the active site of NEST. Moreover, structural and biochemical studies using Cryo-EM or mass spectrometry are needed to reveal the interaction of NEST with inhibitors experimentally.

In summary, our computational modeling and docking analysis provide insights into the different binding interactions of aging and non-aging NTE inhibitors, which may contribute to the different mechanisms of aging and non-aging NTE inhibitors. Therefore, computational modeling might be used to help predict the aging ability of chemicals on NTE in order to predict their potential of inducing delayed neuropathy. 

## Figures and Tables

**Table 1:** Predicted CDOCKER energy of 5 NTE inhibitors and 1 substrate with NEST AlphaFold model by molecular docking.

Ligand	Ligand Type	CDOCKER Energy (kcal/mol)
CBDP	Aging inhibitors	−30.8
PV	Substrate	−29.5
Mipafox	Aging inhibitors	−29.2
Phosphinic acid	Non-aging inhibitors	−25.6
DFP	Aging inhibitors	−21.7
PMSF	Non-aging inhibitors	−19.9

**Table 2:** Interactions of NTE inhibitors with important amino acid residues of NEST. “+” and “−” indicate whether there is a direct interaction (hydrophobic interaction or hydrogen bond) between NEST and ligands.

Regions in NEST	Residues	Aging NTE Inhibitors			Non-Aging NTE Inhibitors		Substrate
		Mipafox	DPF	CBDP	PMSF	Phosphinic Acid	PV
Catalytic dyad	Ser1014	+	+	+	+	+	+
	Asp1134	+	−	+	−	+	−
Oxyanion holes	Gly986	+	−	+	−	+	+
	Gly987	+	+	+	+	+	+
	Arg989	−	−	−	−	+	−
Hydrophobic pockets	Phe1066	−	−	−	+	+	+
	Met1114	−	+	+	+	+	−
	Leu1116	−	+	+	−	+	−
Others	Ile1015	−	+	+	+	−	+
	Gly985	−	−	−	−	+	+
	Trp1039	−	−	−	−	+	−

**Table 3:** The shortest distance between the carbon atoms of the inhibitors and the center point of the benzene ring of NEST Phe1066.

Ligands	Aging NTE Inhibitors			Non-Aging NTE Inhibitors		Substrate
	Mipafox	DPF	CBDP	PMSF	Phosphinic Acid	PV
Distance (Å)	6.0	6.2	5.7	4.8	4.7	4.62

## Data Availability

The data presented in this study are available upon request.
